# Synthesis of Green Engineered Silver Nanoparticles through *Urtica dioica*: An Inhibition of Microbes and Alleviation of Cellular and Organismal Toxicity in *Drosophila melanogaster*

**DOI:** 10.3390/antibiotics11121690

**Published:** 2022-11-23

**Authors:** Mahendra P. Singh, Shabnam Shabir, Aman Singh Deopa, Sanchina Raj Raina, Farkad Bantun, Naif A. Jalal, Noha E. Abdel-razik, Yahya F. Jamous, Maryam S. Alhumaidi, Khadijah A. Altammar, Ahmed Hjazi, Sandeep Kumar Singh, Emanuel Vamanu

**Affiliations:** 1 School of Bioengineering and Biosciences, Lovely Professional University, Phagwara 144411, India; 2 Department of Microbiology, Faculty of Medicine, Umm Al-Qura University, Makkah 24451, Saudi Arabia; 3 Department of Medical Laboratory Technology, Faculty of Applied Medical Sciences, Jazan University, Gizan 82651, Saudi Arabia; 4 National Center of Vaccines and Bio Processing, King Abdulaziz City for Science and Technology (KACST), Riyadh 11564, Saudi Arabia; 5 Department of Biology, College of Science, University of Hafr Al Batin, Hafr Al Batin 31991, Saudi Arabia; 6 Department of Medical Laboratory Sciences, College of Applied Medical Sciences, Prince Sattam bin Abdulaziz University, Al-Kharj 11942, Saudi Arabia; 7 Indian Scientific Education and Technology Foundation, Lucknow 226002, India; 8 Faculty of Biotechnology, University of Agricultural Sciences and Veterinary Medicine, 011464 Bucharest, Romania

**Keywords:** antimicrobial activity, AgNPs, antioxidants, *Drosophila*, green synthesis, *Urtica dioica*

## Abstract

Plant fractions have a diversity of biomolecules that can be used to make complicated reactions for the bioactive fabrication of metal nanoparticles (NPs), in addition to being beneficial as antioxidant medications or dietary supplements. The current study shows that *Urtica dioica* (UD) and biologically synthesized silver nanoparticles (AgNPs) of UD have antibacterial and antioxidant properties against bacteria (*Escherichia coli* and *Pseudomonas putida*) and *Drosophila melanogaster* (Oregon R+). According to their ability to scavenge free radicals, DPPH, ABTS, TFC, and TPC initially estimated the antioxidant potential of UD and UD AgNPs. The fabricated AgNPs were analyzed (UV–Vis, FTIR, EDS, and SEM) to determine the functional groups (alcohol, carboxylic acids, phenol, proteins, and aldehydes) and to observe the shape (agglomerated crystalline and rod-shaped structure). The disc diffusion method was used to test the antimicrobial properties of synthesized Ag-NPs against *E. coli* and *P. putida*. For 24 to 120 h, newly enclosed flies and third instar larvae of *Drosophila* were treated with UD and UD AgNPs. After exposure, tests for biochemical effects (acetylcholinesterase inhibition and protein estimation assays), cytotoxicity (dye exclusion), and behavioral effects (jumping and climbing assays) were conducted. The results showed that nanoparticles were found to have potent antimicrobial activity against all microbial strains tested at various concentrations. In this regard, ethno-medicinal characteristics exhibit a similar impact in *D. melanogaster*, showing (*p* < 0.05) significantly decreased cellular toxicity (trypan blue dye), enhanced biochemical markers (AChE efficacy and proteotoxicity), and improved behavioral patterns in the organism treated with UD AgNPs, especially in comparison to UD extract. The results of this study may help in the utilization of specific plants as reliable sources of natural antioxidants that may have been beneficial in the synthesis of metallic NPs, which aids in the production of nanomedicine and other therapeutic applications.

## 1. Introduction

Nanobiotechnology, including metal nanoparticles (NPs), has attracted great interest owing to its reducing character and broad application spectrum in practically each domain of research and technology, especially biological engineering [1]. These tiny particles differ in attributes from a chemical aspect with the same composition because of their high surface-to-volume ratio [2]. Metal NPs are currently very significant due to their optical characteristics, catalytic activity, electrical properties, magnetic activity, antioxidant activity, and antibacterial activity [3]. In medicine and pharmacy, NPs are used in a variety of innovative ways, such as in medical imaging, filters, nanocomposites, drug carriers, and compositions, to treat various diseases, including cancer [4,5,6,7]. In data analysis for the identification of proteins, antibodies, and cancer cells, gold NPs have been employed [8,9]. Even against infectious organisms, including *Pseudomonas putida*, *Escherichia coli*, *Syphillis typhus*, *Staphylococcus aureus*, and *Vibria cholera*, AgNPs are also recognized as antimicrobial agents [10]. However, apart from silver and gold NPs, the literature also discussed NPs made of copper oxide (CuO NPs), zinc oxide (ZnO NPs), iron (I NPs), copper (Cu NPs), nickel oxide (NiO NP), and NPs made of iron hydroxide (I OOH) and iron oxide (IO NPs) [11,12,13,14].

NPs synthesized chemically frequently need harmful stabilizing and reducing chemicals (ethylene glycol, hydrazine hydrate, *dimethylformamide*, and sodium borohydride) [15]. Natural techniques of synthesis involving microbes and biological systems are required for the fabrication of nanomaterials for biomedical purposes [16]. A technique known as biogenic synthesis or green synthesis has been acknowledged as an environmentally friendly method for producing a variety of nanomaterials, including hybrid materials and therefore bioinspired materials, in addition to metal/metal oxide nanoparticles. NPs can be synthesized biologically by combining metal cations with living creatures, plant fractions, algae, yeasts, bacteria, lichens, and fungi [17,18]. In contrast to those produced chemically, the NPs produced through biosynthesis are more stable and less harmful [19]. The extract components, which contain a variety of bioactive substances, such as trace metal ions, terpenoids, alkaloids, flavonoids, polyphenols, vitamins, saponins, carotenoids, and biological catalysts (enzymes), are essential for the formation of NPs because they act as potent stabilizing agents, reducing agents, or precursor molecules for the formation of NPs [20]. There are two steps in the synthesis of NPs. The metal ions are reduced in the first step, and then, colloidal nanoparticles clump together to form oligomeric clusters [21].

The key challenges in the biogenic synthesis of nanoparticles are acquiring the appropriate shape, size, and crystallinity of the particles in the solution phase [22]. Numerous variables, including temperature, pH, and incubation or reaction time, have often been considered to manage the synthesis process [23]. In addition to their ability to produce AgNPs, natural extracts have been shown in numerous studies to possess outstanding antioxidant activity that is superior to that of substrates as shown in Table 1 [24]. This activity is thought to be caused by the extract components preferentially solubilizing on nanoparticle surfaces [25]. AgNPs mediated by extracts have also demonstrated anticancer properties [26]. AgNPs generated from *Azadirachta indica* leaf and *Calligonum comosum* root fractions have been shown to be cytotoxic to HepG2, LoVo, and MDA-MB231 cell lines [27]. The AgNPs fabricated utilizing the *M. citrifolia* root fraction resulted in the complete demise of the HeLa cell lines [28]. In addition, extracts from *Datura inoxia* [29] and *Alternanthera tenella* [30] seemed helpful in the formation of AgNPs that inhibited the proliferation of MCF-7 cell lines (human breast cancer). Recent studies have demonstrated the effectiveness of the *C. sativa* leaf fraction in mediating the synthesis of AgNPs and their antibacterial potential against a few harmful bacteria, including *P. fluorescens*, *S. aureus*, *K. pneumoniae*, and *E. coli* [31].

Since ancient times, *U. dioica* has been a staple herbal treatment [32]. The edible section of nettle plants is rich in a range of compounds, including polyphenols, quercetin, reducing sugars, alkaloids, and vitamins [33]. By reducing and chelating metal ions, scavenging free radicals, reducing lipids, and interacting favorably with antioxidant enzymes, these plants have powerful antioxidant potential [34]. UD has undergone significant research and has demonstrated good outcomes in rat colon carcinogenesis [35], the management of prostate enlargement [36], prevention of hypercholesterolemia [37] and hyperglycemia [38].

**Table 1:** Synthesis of green silver nanoparticles using various plant extract and its applications.

S. No.	Plants	Plant Part Used	Size (nm)	Shape	Properties	References
1.	*Moringa oleifera*	Leaves	12	Rectangle	Antimicrobial and anticancer agents	[39]
2.	*Azadirachta indica*	Leaves	19–20	Triangle	Antibacterial, skin healing, and antifungal	[40]
3.	*Ocimum tenuiflorum*	Leaves	49–50	Cuboidal	Antibacterial and antioxidant	[40]
4.	*Lansium domesticum*	Fruit	11–31	Spherical	Wound healing potential and anti-aging	[41]
5.	*Phytolacca decandra*	Whole plant	89.96	Spherical	Antioxidant and anti-parasitic	[42]
6.	*Vitex negundo*	Leaves	19–20	Cubic	Antibacterial activity	[43]
7.	*Nelumbo nucifera*	Root	15.9	Polydispersed	antimicrobial and anti-diarrheal	[44]
8.	*Cucurbita maxima*	Petals	20	Crystals	Antimicrobial	[39]
9.	*Calotropis g* *ig* *antea*	Latex	6–29	Spherical	Antidote for snake bite	[45]
10.	*Carica papaya*	Leaves	49–249	Spherical	Antibacterial activity	[46]

In this study, silver nanoparticles synthesized from *Urtica dioica* leaf extract by green synthesis and its modulatory effects in *Drosophila melanogaster* were reported for the first time. The basic cognitive and preventive effects of UD have been documented in the literature. The current study demonstrates the amelioration of UD extract and biologically synthesized silver nanoparticles (AgNPs) of UD against bacteria (*Escherichia coli* and *Pseudomonas putida*) and *Drosophila melanogaster* (Oregon R+). The fabricated NPs were analyzed through FTIR, SEM, EDX, and UV-visible techniques. The disc diffusion method was employed to test the antimicrobial properties of synthesized AgNPs against *E. coli* and *P. putida*. In addition, using a nontarget in vivo model of *Drosophila*, we aimed to study the modulatory effects at the cellular and neurological levels. We also examined its organismal effects on *D. melanogaster*, which has been shown to be a useful model for neurodegenerative disorders such as Parkinson’s disease (PD).

## 2. Results

The subsections below describe the findings of the experiments and assessments used to evaluate the potential of UD and UD-AgNPs in microorganisms (*E. coli* and *P. putida*) and *D. melanogaster*.

### 2.1. Analytical Assays

#### 2.1.1. The Antioxidant Efficacy of UD and UD-AgNPs

**Figure 1:**
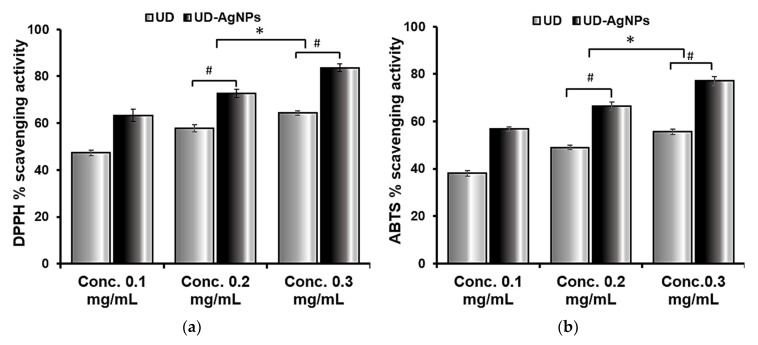
The antioxidant activity of AgNPs produced from *U. dioica* extract was measured and compared to the DPPH (**a**) and ABTS (**b**) assays. At various concentrations, the antioxidant capacity was measured (0.1, 0.2, and 0.3 mg/mL). The radical scavenging abilities of DPPH and ABTS were dose dependent, implying that as the concentration of AgNPs increased; similarly, the scavenging activities increased against both radicals (the antioxidant potential for DPPH and ABTS was 63.3–83.5% and 56.7–77.1%, respectively). The data are shown as the mean ± SD (*n* = 3). Statistical significance was ascribed as * *p* < 0.05 (intergroup) and # *p* < 0.05 (intragroup) compared with and 0.1 mg/mL of the respective groups.

The DPPH assay technique is based on the antioxidant ability to scavenge DPPH radicals. The maximal UV-vis absorption of DPPH is between 515 and 520 nm. The organic nitrogen radical combines with hydrogen/electron donor molecules, resulting in a brownish or yellow radical solution with decreased absorbance. The in vitro reductive and antiradical activities of UD and UD-AgNPs are beneficial for future antioxidant studies. As illustrated in Figure 1, UD-AgNPs had a stronger radical scavenging potential than the UD extract in DPPH. The primary factor responsible for the antioxidant capacity of fractions is the redox potential of phenolic components, which function as reducing agents. As previously mentioned, DPPH can take both an electron and a hydrogen radical, which turn it into a stable diamagnetic molecule.

ABTS is an unstable, free radical that is employed to study the antioxidant abilities of hydrophilic and hydrophobic antioxidants present in nutraceutical fractions. The radical scavenging ability of ABTS in the samples obtained was found to be highest in UD-AgNPs, followed by UD. The maximal UV–Vis absorption of ABTS is 415. These findings suggest that biosynthesized silver nanoparticles of UD have a greater potency in scavenging ABTS free radicals, as well as enhanced antiradical and antioxidant ability.

#### 2.1.2. EC_50_ Calculation Using Statistical Tools

The EC_50_ value is an important characteristic for analyzing antioxidant potential and might be used to calculate the antioxidant activity of various substances. The EC_50_ can be estimated by plotting data from an appropriate curve or by using a regression model with multiple components on the data. EC_50_ can be computed using a variety of models. Lower EC_50_ values suggest more radical scavenging activity.

**Figure 2:**
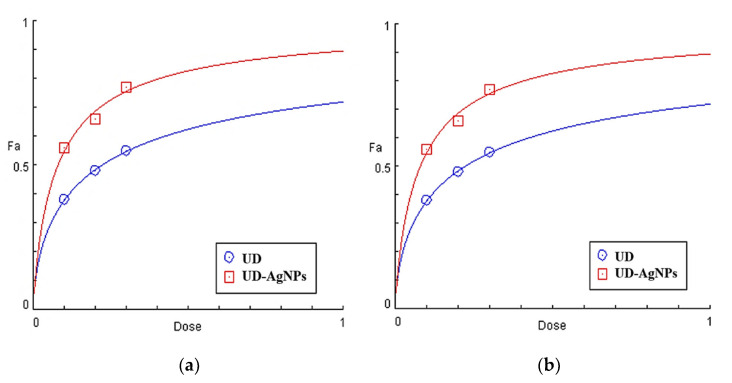
Dose–response profiles of the calculated EC_50_ (mg/mL) of *Urtica dioica* (UD) and biosynthesized nanoparticles of UD (UD-AgNPs) in the DPPH (**a**) and ABTS (**b**)-free radical scavenging tests.

UD-AgNPs were shown to have enhanced antioxidant potential due to a higher total phenolic content and flavonoid concentration, with EC_50_ values of 0.05 mg/mL and 0.12 mg/mL for UD in DPPH scavenging. The EC_50_ values for UD-AgNPs are 0.07 mg/mL and 0.22 mg/mL for UD in ABTS radicals, respectively, as shown in Figure 2 and Table 2.

#### 2.1.3. Flavonoid and Phenolic Potential of UD and Biosynthesized AgNPs of UD

After AgNPs synthesis, the TFC and TPC of the UD extract and UD-AgNPs supernatant were evaluated, and the results were analyzed based on quercetin and gallic acid equivalents, respectively. The total phenol and total flavonoid decreases in the extracts were determined to quantify the number of flavonoids and phenols employed in the fabrication of AgNPs or associated with their surfaces.

**Figure 3:**
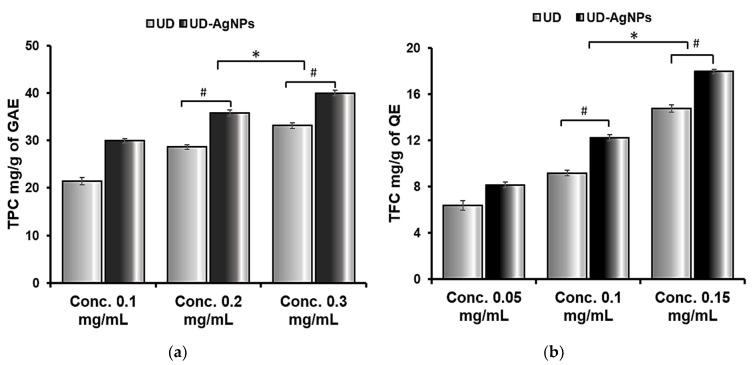
Total phenolic (**a**) and flavonoid (**b**) contents of *Urtica dioica* (UD) and biosynthesized silver nanoparticles of UD (UD-AgNPs). The data are shown as the mean ± SD (*n* = 3). Statistical significance was ascribed as * *p* < 0.05 (intergroup) and # *p* < 0.05 (intragroup) compared with and 0.1 mg/mL of the respective groups.

In terms of total flavonoid content (TFC), biosynthesized AgNPs of UD (17.94 mg (QE)/g) were determined to be the highest, followed by UD extract (14.6 mg (QE)/g). In addition, the same was observed for the total phenolic content (TPC); UD (33.12 mg (GAE)/g) had a lower flavonoid concentration than UD-AgNPs (40.00 mg (GAE)/g) (Figure 3 and Table 3).

#### 2.1.4. Characterization Techniques of Biosynthesized Silver Nanoparticles (AgNPs)

In determining the morphology, size, structure, shape, surface chemistry, dispersity, surface area, and surface charge of AgNPs, characterization is a fundamental step. Several methods are implemented to characterize AgNPs, which are given below.

#### 2.1.5. UV–Vis Spectroscopy

**Figure 4:**
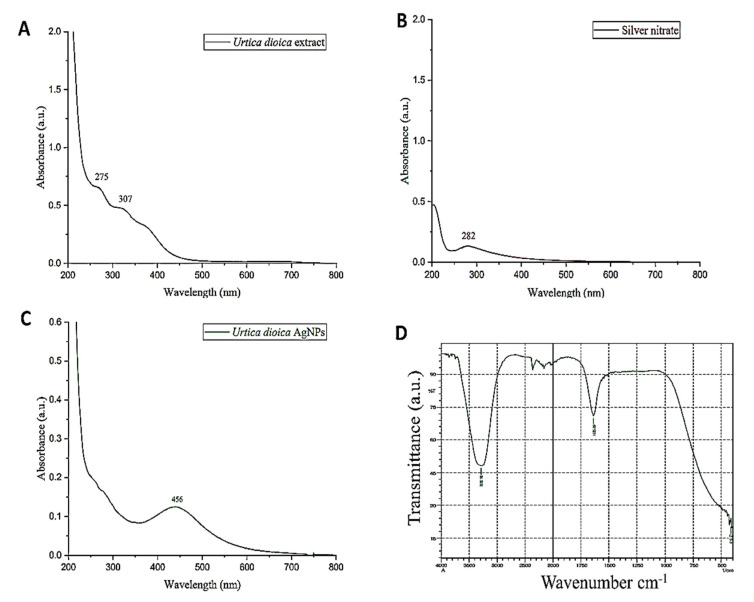
UV–visible spectra of *Urtica dioica* extract (**A**), silver nitrate (**B**), and biosynthesized nanoparticles of UD (**C**) in the absorption spectrum of 200–800 nm. The functional groups of *Urtica dioica* leaf extract responsible for the formation and stability of silver nanoparticles (UD-AgNPs) were investigated using FTIR (**D**) with a scan range of 400–4000 cm^−1^.

The biosynthesis of AgNPs using UD in an aqueous medium was evaluated by taking the absorption spectrum in the 200–800 nm wavelength range. When silver nitrate solution was added to the UD leaf fraction, it turned dark brown in color, indicating the synthesis of AgNPs, whereas there was no change in color in the absence of the plant fraction (Figure 4A–C). A distinct, intense, and broad (surface plasmon resonance) peak at 456 nm was observed in the UV–Vis spectra, confirming the synthesis of AgNPs. Previous research suggested that AgNPs have an SPR peak between 410 and 460 nm, which could be attributed to spherical NPs.

#### 2.1.6. Fourier-Transform Infrared Spectroscopy (FTIR)

Fourier-transform infrared technology is a technique that helps in the qualitative analysis of nanomaterials. It determines the functional groups (alcohols, phenols, alkanes, and ketones) present in the synthesized silver nanoparticles (AgNPs) of UD. The presence of functional groups can be observed from the peaks formed in the FTIR spectrum (Figure 4D). The spectra represent the peaks at 420.5 cm^−1^, 1635.69 cm^−1^, and 3282.95 cm^−1^. The absorbance spectra fall in the range of 400–500 cm^−1^. Two other peaks were monitored in the spectra that are elongated U-shaped. These two peaks represented two functional groups at two different wavenumbers. The adsorption peak at 3282.95 cm^−1^ represents the hydroxyl group (OH) of the compound class alcohol. Another adsorption peak at 1635.69 cm^−1^ represents the C=C group of compound class alkene. The peaks formed in the FTIR spectrum are due to the presence of flavonoids and phenols in the leaf fraction of the *U. dioica* plant. These phenols and flavonoids are responsible for the reduction of Ag^+^ ions and the formation of AgNPs.

#### 2.1.7. Scanning Electron Microscopy (SEM)

**Figure 5:**
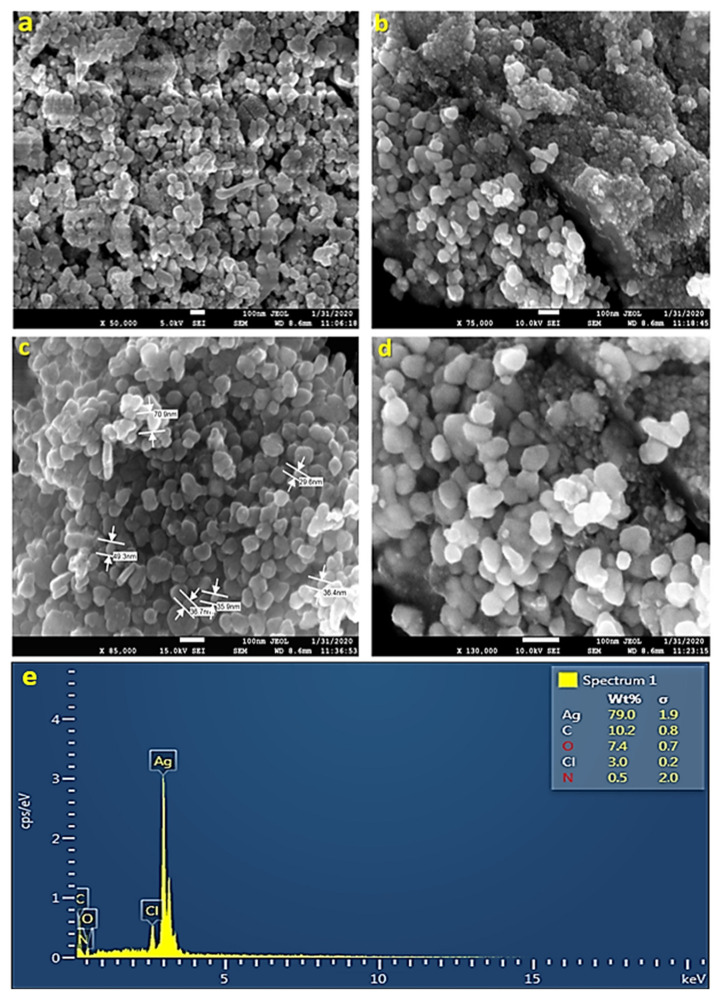
SEM-EDX spectra of biosynthesized silver nanoparticles. SEM images (**a**–**d**) of the AgNPs demonstrate that their spherical shape ranges from 29 to 70 nm in size. An elemental analysis of AgNPs was confirmed by EDX (**e**), which reveals that 79% of the material is made up of Ag. A strong peak signal was observed at 3 KeV, which is characteristic of metallic AgNPs absorption.

Scanning electron microscopy helps in the quantitative and qualitative analysis of nanomaterials. It determines the shape and size of AgNPs. SEM works on the principle of scanning the nanoparticle sample under an electron beam of high energy. The sample is placed under vacuum conditions. SEM provides images with high resolution, which makes it a useful and widely employed instrument in determining the size and shape of NPs. The SEM images revealed that the shape of some of the AgNPs obtained was spherical; some were oval, while others were irregular in shape. Figure 5a,b shows the size of AgNPs, which varies from 29 to 70 nm. The irregular shape of AgNPs could have been because of the temperature, pH, and concentration of AgNO_3_ (Figure 5a–d).

#### 2.1.8. Energy Dispersive X-ray Spectroscopy (EDS)

EDS is a technique that helps in the chemical analysis of nanoparticles. Energy-dispersive X-ray spectroscopy monitored the X-rays generated by the sample (UD-AgNPs), which was placed under an electron beam. The energy of the X-rays forms the peaks in the spectrum. Analysis of AgNPs through EDS is performed in the energy range of 0–20 keV. The spectra show a strong indication of silver in the synthesized sample at 3 keV. From the EDS results, it was observed that the concentration of silver in the nanoparticles was 79% (Figure 5e). The other peaks obtained showed the presence of other compounds in the nanoparticle sample, such as carbon (10.2%), oxygen (7.4%), chlorine (3.0%), and nitrogen (0.5%). The presence of carbon might have been due to contamination all over the place, whereas the presence of oxygen, chlorine, and nitrogen indicated that leaves constitute some organic components as well.

### 2.2. The Bactericidal Potential of Green-Engineered AgNPs against E. coli and P. putida

**Figure 6:**
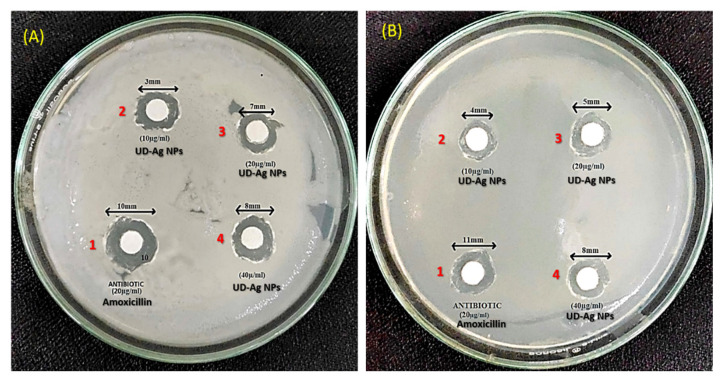
Antibacterial potential of AgNPs fabricated using the aqueous leaf fraction of *U. dioica* against *Escherichia coli* (**A**) and *Pseudomonas putida* (**B**) bacterial strains. Note: 1 = Antibiotic (Amoxicillin 20 µg/mL), 2 = UD-AgNPs (10 µg/mL), 3 = UD-AgNPs (20 µg/mL), and 4 = UD-AgNPs (40 µg/mL).

The silver nanoparticles (AgNPs) were tested against *E. coli* and *P. putida* at different concentrations via the disc diffusion method by determining the zone of inhibition (Figure 6A,B). From Table 4 shown below, it can be observed that the diameter for the zone of inhibition is increased by increasing the concentration of the AgNPs. The control that was taken is amoxicillin, which is an antibiotic that possesses the maximum diameter in the table. Hence, the AgNPs extracted from nettle leaves possess antimicrobial or bactericidal properties as they were tested against an antibiotic. In all the above mentioned tabular cases, zones of inhibition are observed.

### 2.3. Cellular Assays

Cytotoxicity of AgNO_3_ and amelioration through bioactive compounds of UD and UD-AgNPs evaluated by a dye exclusion test (Trypan Blue) in tissues of treated *Drosophila*.

**Figure 7:**
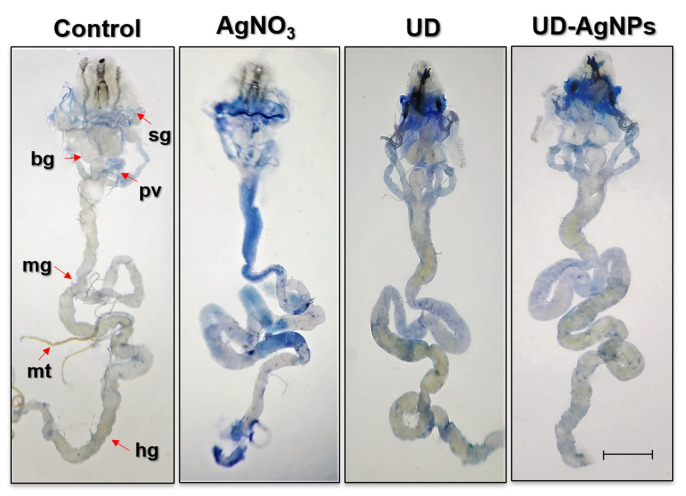
Dye exclusion test through trypan blue staining in dissected third instar larvae (Seventy-two hours old) of *D. melanogaster* (Oregon R+) exposed to AgNO_3_, UD, and UD-AgNPs for 48 h. Note: bg = brain ganglia, pv = proventriculus, sg = salivary glands, mt = Malpighian tubules, mg = midgut, and hg = hind gut. The bar represents 100 μm. AgNO_3_ = silver nitrate, UD = *Urtica dioica,* and UD-AgNPs = biosynthesized silver nanoparticles of *Urtica dioica*.

We evaluated trypan blue staining in *D. melanogaster* larval tissues, as shown in Figure 7, to determine whether AgNO_3_ exposure results in tissue damage. Larvae exposed to AgNO_3_ exhibited 45% blue staining in their tissues (midgut, brain ganglia, gastric caeca, and salivary gland). UDCA, biosynthesized AgNPs of UD, and the control showed far less blue staining than the AgNO_3_ group in the abovementioned tissues.

### 2.4. Biochemical Assays

#### 2.4.1. Biosynthesized AgNPs of UD Improved AChE Activity in *D. melanogaster*

In this research, it was observed that when the larvae were treated with AgNO_3_ (90.3 ± 0.93 × 10^4^ moles/min/g) for 24 h, there was a statistically significant (*p* < 0.001) reduction in AChE levels in comparison to the untreated/control (100.0 ± 1.65 × 10^4^ moles/min/g) group. The AChE levels in the UD (106.8 ± 0.93 × 10^4^ moles/min/g) and biosynthesized AgNPs of UD (108.8 ± 0.98 × 10^4^ moles/min/g) groups improved significantly as well. UD-AgNPs had significantly higher AChE levels than the other groups.

**Figure 8:**
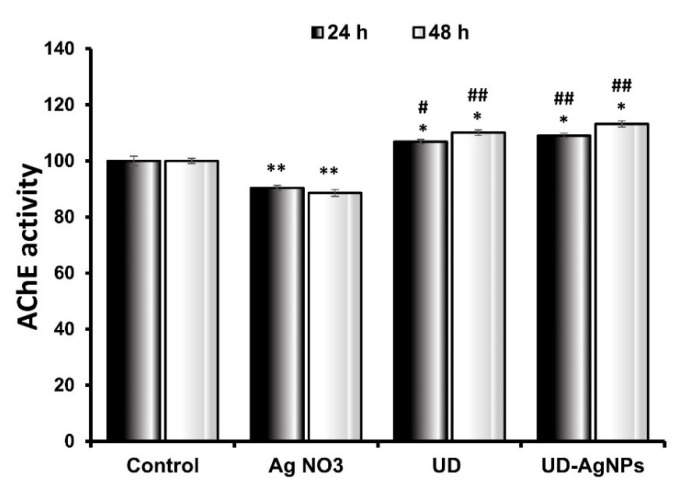
Acetylcholinesterase activity in *Drosophila* third instar larvae exposed to AgNO_3_, UD, and UD-AgNPs for 24 h and 48 h. The data are shown as the mean ± SD (*n* = 3). Significance is indicated as * *p* < 0.05 and ** *p* < 0.01 vs. control. # is ascribed as significance at *p* < 0.05 and ## *p* < 0.01 compared with AgNO_3_. AgNO_3_ = silver nitrate, UD = *Urtica dioica*, and UD-AgNPs = biosynthesized silver nanoparticles of *Urtica dioica*.

After 48 h, when compared to control (100.1 ± 1.16 × 10^4^ moles/min/g) larvae, AgNO_3_ (88.6 ± 1.18 × 10^4^ moles/min/g)-treated organisms showed the greatest suppression of AChE levels. The AChE levels were significantly higher in the UD (111.0 ± 0.9 × 10^4^ moles/min/g) and UD-AgNPs (119.1 ± 1.16 × 10^4^ moles/min/g) groups than in the control treatment group (Figure 8). The AChE levels in the UD-AgNPs were substantially greater than those in the other groups.

#### 2.4.2. Increased Protein Content in *Drosophila* Treated with UD and UD-AgNPs after 24 and 48 h

**Figure 9:**
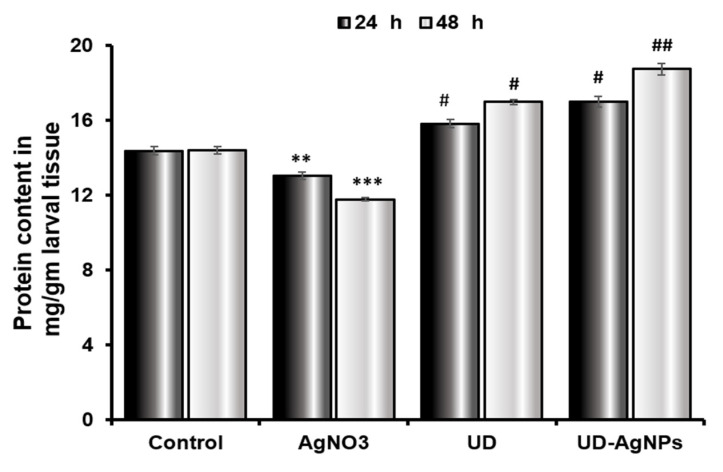
The protein concentration in third-instar *Drosophila melanogaster* (Oregon R+) larvae treated with AgNO_3_, UD, and UD-AgNPs for 24 h and 48 h. The results are the mean ± SD of three replicates of the same experiments. Significance is ascribed as ** *p* < 0.01, and *** *p* < 0.001 vs. control. # = significance at *p* < 0.05, and ## *p* < 0.01 compared with AgNO_3_. AgNO_3_ = silver nitrate, UD = *Urtica dioica*, and UD-AgNPs = biosynthesized silver nanoparticles of *Urtica dioica*.

The protein content in the tissues of AgNO_3_-exposed *Drosophila* third instar larvae significantly decreased (*p* < 0.001) after exposure. The protein content after 24 h in the larvae was decreased in the AgNO_3_ group (13.03 ± 0.21 mg/mL) compared to the control group (14.37 ± 0.23 mg/mL). Silver nanoparticles synthesized by the UD group (16.99 ± 0.30 mg/mL) excelled in the UD group (15.82 ± 0.20 mg/mL). In comparison to the untreated/control group (14.41 ± 0.19 mg/mL) after 48 h, the UD-AgNPs (18.74 ± 0.31 mg/mL) treatment enhanced the level of protein in the larvae, followed by UD (16.99 ± 0.13 mg/mL) and AgNO_3_ (11.78 ± 0.09 mg/mL) treatment (Figure 9).

### 2.5. Behavioral Assays

#### 2.5.1. AgNO_3_ Affects Locomotor Behavior (Climbing Ability) in *Drosophila*

**Figure 10:**
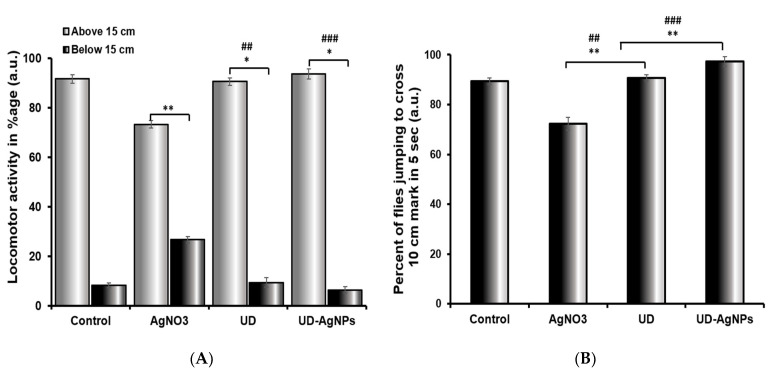
Climbing (**A**) and jumping (**B**) activity of *Drosophila melanogaster* (Oregon R+) flies exposed to AgNO_3_, UD, and UD-AgNPs for 120 h. The data are shown as the mean ± SD (*n* = 3). Significance is ascribed as * *p* < 0.05 and ** *p* < 0.01 vs. control. ## ascribed as significance at *p* < 0.01 and ### *p* < 0.0001 compared with AgNO_3_. AgNO_3_ = silver nitrate, UD = *Urtica dioica*, and UD-AgNPs = biosynthesized silver nanoparticles of *Urtica dioica*.

In comparison to the untreated/control group, the UD-AgNPs-treated flies displayed the greatest climbing ability in 30 s. Flies found it challenging to climb the cylinder walls in AgNO_3_-exposed *Drosophila*, where the greatest reduction was observed. The different groups show variable degrees of progress in their climbing abilities. Among all groups, UD-AgNPs (6.33%) had the lowest reduction, followed by UD (9.33%), control (8.63%), and AgNO_3_ (26.66%). An unpaired Student’s *t*-test was employed to compare the mean ± SEM to determine whether any differences were statistically significant. *p* < 0.001 was assigned as the level of significance (Figure 10).

#### 2.5.2. Significant Variation in Jumping Ability of UD-AgNPs-Exposed Organisms

The jumping behavior of the flies treated with AgNO_3_ was dramatically reduced (27.68%) when compared to the control. The various groups showed varying degrees of improvement in their jumping abilities. The UD-AgNPs (2.66%) showed the least reduction, preceded by UD (9.32%), the control (10.64%), and AgNO_3_, which demonstrated the greatest reduction. An unpaired Student’s *t*-test was used to compare the mean ± SEM to detect statistically significant differences. *p* < 0.001 was assigned as the significance level (Figure 10B).

## 3. Discussion

**Figure 11:**
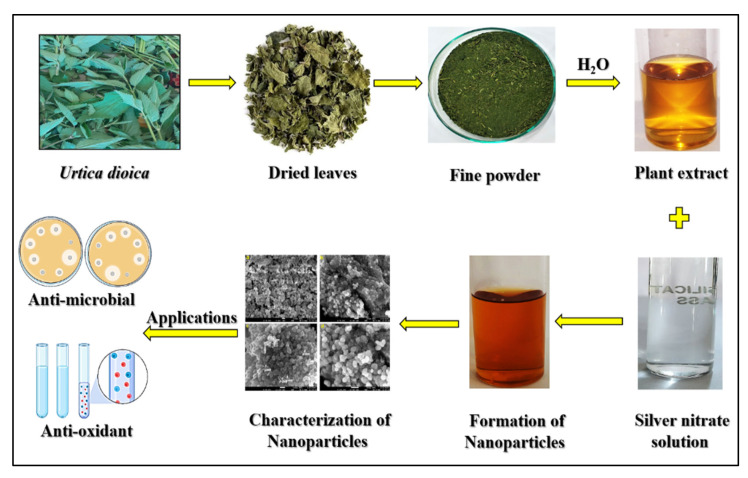
Graphical representation and applications of green synthesized silver nanoparticles through *Urtica dioica*.

The present research demonstrated the biosynthesis, characterization, and applications of silver nanoparticles (AgNPs) in microorganisms (*E. coli* and *P. putida*) as well as the nontarget organism *D. melanogaster* at the cellular, neurological, and organismal levels for the first time. AgNPs were synthesized biologically from *Urtica dioica* (UD) leaves, the extract of which acts as a reducing agent that reduces Ag+ ions from silver nitrate (AgNO_3_). The AgNPs were synthesized in the form of dark brown pellets, which were converted into powdered form and utilized for characterization purposes (Figure 11).

The UD leaf fraction contains a variety of secondary metabolites, including amino acids, proteins, reducing sugars, polysaccharides, vitamins, and enzymes, which may act as metal ion reductants or scaffolds to promote the synthesis of Ag nanoparticles in solution [47]. Moreover, the mechanism behind silver bio reduction was thought to initially involve electrostatic interactions trapping Ag ions on the protein surface in the plant fraction (recognition process). Aldehyde groups in plant fractions are involved in the conversion of Ag ions into metallic Ag nanoparticles. The distinct functional groups C=N and -C=O denote amide I of polypeptides, which oversees capping ionic compounds into metallic nanoparticles. Proteins then decrease silver ions, causing secondary structural modifications and the production of silver nuclei. Molecular investigations on the production of Ag crystals have revealed a complicated system that grows by subsequent reduction of Ag ions and aggregation at nuclei [48]. The physiochemical properties required presently included the morphology, size, and chemicals present, as well as the chemical compounds or functional groups present in the synthesized AgNPs [49]. For this research, the characterization techniques implemented were UV–vis, SEM, EDS, and FTIR.

Characterization is an important step in determining the morphology, size, structure, shape, surface chemistry, dispersity, surface area, and charge of AgNPs [50]. Several approaches are being used to characterize AgNPs, which are listed below. UV–vis spectrophotometry is a widely used technique for characterizing fabricated nanoparticles, and it is also often used to determine the stability and formation of Ag nanoparticles. The redshift in the UV–vis spectrum of UD-AgNPs is related to the commencement of the nanocrystal growth phase, which results in the production of more and larger particles without aggregation [51]. This could possibly be due to the presence of fraction constituents on the NPs acting as capping agents. It is important to observe that these findings are remarkably comparable to previous research [52]. In general, the significantly rapid color change and acute absorbance intensity seen in the first minutes of the process reveal the capability of UD extract in ultrafast nanoparticle synthesis. Numerous studies have demonstrated that AgNPs exhibit absorption bands in the UV–Visible spectral region of 200–800 nm and are employed for NP characterization with a spectrum of 2–100 nm [53]. A distinct, intense, and broad (surface plasmon resonance) peak at 456 nm was observed in the UV–Vis spectra, confirming the synthesis of AgNPs. Previous research suggested that AgNPs have an SPR peak between 410 and 460 nm, which could be attributed to spherical nanoparticles [54].

The Fourier-transform infrared technique is used to study the surface characteristics of metal nanoparticles and ascertain whether functional elements are involved in NP formation. Moreover, the catalytic interaction between the enzyme and substrate has been studied, as well as the verification of bioactive substances covalently bound to silver [55]. By creating a sample diffraction pattern and depicting absorption and transmission, the resulting spectrum identifies UD-AgNPs. FTIR is an appropriate, beneficial, affordable, and simple tool for investigating the function of biomolecules in the conversion of AgNO_3_ to Ag [56]. The results of this study represent the peaks at 420.5 cm^−1^, 1635.69 cm^−1^, and 3282.95 cm^−1^. The absorbance spectra fall in the range of 400–500 cm^−1^. Two other peaks were observed in the spectra that are elongated U-shaped. These two peaks represented two functional groups at two different wavenumbers. The adsorption peak at 3282.95 cm^−1^ represents the hydroxyl group (OH) of the compound class alcohol. Another adsorption peak at 1635.69 cm^−1^ represents the C=C group of compound class alkene. The peaks formed in the FTIR spectrum are due to the presence of phenols and flavonoids in the leaf fraction of UD. These phenols and flavonoids are involved in the reduction of Ag^+^ ions and the fabrication of AgNPs [57]. SEM is a surface optical technique that can determine distinct particle forms, shapes, size distributions and surface morphologies of fabricated NPs at the micro (10^−6^) and nano (10^−9^) scales [58]. A high-energy electron beam is used in SEM to scan across the AgNPs sample’s surface, and backscattered electrons are then detected to provide sample properties. SEM images showed that some of the AgNPs obtained were spherical, while others were oval, and the sizes of AgNPs varied from 29 to 70 nm. The irregular shape of AgNPs could have been because of the temperature, pH, and concentration of AgNO_3_ [59]. The elemental composition of a AgNPs sample can be determined using EDX spectroscopy in conjunction with SEM. The EDS technique uses an X-ray detector to quantify the ratio of the number of discharged X-rays to their energy to identify the X-rays generated by the sample during electron beam irradiation [60]. From the EDS results, it was observed that the concentration of silver in the nanoparticles was 79%. The other peaks obtained showed the presence of other different compounds in the nanoparticle sample, such as carbon (10.2%), oxygen (7.4%), chlorine (3.0%), and nitrogen (0.5%). The presence of carbon might have been due to contamination all over the place, whereas the presence of oxygen, chlorine, and nitrogen indicated that leaves constitute some organic components as well.

DPPH and ABTS free radical scavenging assays are commonly used to assess the antioxidant capabilities of AgNPs [61]. At various concentrations, the antioxidant capacity was measured (0.1, 0.2, and 0.3 mg/mL). The radical scavenging abilities of DPPH and ABTS were dose-dependent, implying that, as the concentration of AgNPs increased, the scavenging activities increased against both radicals (the antioxidant potential for DPPH and ABTS was 63.3–83.5% and 56.7–77.1%, respectively). The most well-known nanoparticles are generated from plant fractions, and the antioxidant capability of Ag nanoparticles is frequently compared to that of the plant fraction itself [62]. Some research revealed increased antioxidant activity in AgNPs, whereas others showed the opposite; hence, the results are contradictory. Both outcomes are possible given the large number of studies reporting both kinds of results. In general, the antioxidant effects of AgNPs are determined by the chemical content of the fraction and typically improve as the concentration of AgNPs increases [63]. The nanoparticles exhibit strong scavenging when the extract contains phenolic chemicals and flavonoids.

Since ancient times, Ag has been widely employed as a treatment for a variety of ailments. Before the invention of antibiotics, Ag was used as an antiseptic for the healing of burns and open wounds [64]. To check the effects of AgNPs through green synthesis on bacteria, an experiment on *E. coli* and *P. putida* was conducted, and the effects of AgNPs on both bacteria were studied. The criteria that are mainly focused on were the zone of inhibition at different concentrations of AgNPs, and their effectiveness compared to antibiotics. Different-sized zones of inhibition were observed at different concentrations of AgNPs. Ag nanoparticles have different inhibitory impacts on Gram-positive and Gram-negative bacteria; rather, they favor one over the other. Gram-positive bacterial strains have been shown in some studies to be more sensitive to Ag nanoparticles than Gram-negative bacterial strains, but other investigations have found the opposite [65]. Ag nanoparticles are positively charged, whereas bacterial cell membranes are negatively charged. When positively charged Ag nanoparticles build up on negatively charged membranes, structural changes take place that increase the permeability of the bacterial cell membrane [66]. Therefore, uncontrolled trafficking through the cytoplasmic membrane results in cell death. Ag nanoparticles may harm the genetic material within the bacterial cell by contacting it, which hinders transcription and translation [67]. The Trypan blue (dye exclusion) test in *Drosophila* larval tissues was used to investigate the cytotoxicity of UD, AgNO_3,_ and biosynthesized AgNPs of UD. The results showed that larvae exposed to AgNO_3_ exhibited 45% blue staining in their tissues (midgut, brain ganglia, gastric caeca, and salivary glands). UDCA, biosynthesized AgNPs of UD, and the control showed far less blue staining than the AgNO_3_ group in the abovementioned tissues. This finding is supported by a prior study that revealed that plant constituents dramatically increased cell viability. Due to the activation of antioxidant defense systems or the existence of bioactive components that quench free radicals, UD-AgNPs have protective properties [68].

To further understand the harmful effects of AgNO_3_, which raise cellular pro-oxidant levels and enable polypeptides to undergo oxidative posttranslational alterations, biochemical tests were performed after treatment for 24 and 48 h [69]. The protein content in the tissues of AgNO_3_-exposed *Drosophila* third instar larvae was significantly reduced (*p* < 0.001) after exposure. The protein content of the larvae after 24 h was reduced in the AgNO_3_ group compared to the untreated/control group. Silver nanoparticles synthesized by the UD group excelled the UD group. In comparison to the control group after 48 h, the UD-AgNPs treatment enhanced the level of protein in the larvae, followed by UD and finally AgNO_3_ treatment. This result is in accordance with an earlier study, which showed that various plant-mediated nanoparticles increased and maintained the protein concentration in organisms [70].

A fundamental cholinergic system enzyme called acetylcholinesterase (AChE) regulates various physiological activities, such as memory and locomotion [71]. It stops cholinergic neurotransmission between synapses by hydrolyzing acetylcholine into choline and acetate [72,73]. A significant statistical (*p* < 0.001) decrease in AChE activity was seen in this study when the larvae were exposed to AgNO_3_ for 24 h compared to the control/untreated larvae. AChE levels in the UD and biosynthesized AgNPs of UD groups improved significantly as well compared to the control. UD-AgNPs had significantly higher AChE levels than the other groups. After 48 h, when compared to control larvae, AgNO_3_-treated organisms showed the greatest suppression of AChE levels. In comparison to the untreated/control treatment group, AChE levels were considerably greater in the UD and UD-AgNPs groups. The AChE levels in the UD-AgNPs were substantially greater than those in the other groups. AChE levels were found to be increased by green synthesized NPs and AChE inhibition in AgNO_3_-exposed organisms, which is consistent with earlier findings [74,75].

The behavior of an organism reveals its normal physiological activities [76,77]. From this perspective, an organism’s climbing and jumping behaviors reflect its physiological state [78]. In this context, AgNO_3_-induced toxicity may be indicated by a high rate of locomotor deficits as determined by the climbing experiment. Fly locomotor impairments cause them to stay at the bottom of the plastic cylinder, which suggests that they lack regular leg coordination. This phenotypic manifestation has previously been connected to the increased energy needs of the mitochondria-rich muscles used for walking and flying. Although speculative, an uncoupled mitochondrial mechanism may be the origin of the same underlying problems that result in severe complex I inhibition. Interestingly, UD-AgNPs successfully stopped the progression of the flies’ locomotor dysfunctions, suggesting that they might be able to protect themselves by refilling the dopaminergic reserve at the mitochondrial level. This finding confirms previous research that showed a direct link between dopamine depletion and locomotor impairment [79]. The significant decrease in jumping behavior in exposed organisms, which was followed by a decrease in AChE activity, showed the harmful effects of AgNO_3_ on the organism. As a sign of AChE activity suppression, poor locomotor function has previously been noted. Fly jumping ability was increased by UD and UD synthesized AgNPs [80]. The greatest jumping activity was observed in UDNPs, followed by UD, the control, and AgNO_3_.

Taken together, the current study demonstrated the comparative efficacy of UD and UD-AgNPs in microorganisms (*E. coli* and *P. putida*), as well as the nontarget organism *D. melanogaster* at the cellular, neurological, and organismal levels. The fabricated AgNPs were identified by UV–Vis, SEM, EDS, and FTIR techniques to be spherical in shape and range in size from 20 to 70 nm. We conclude that short-term dietary administration of UD-AgNPs to *Drosophila* has the potential to alleviate oxidative stress due to its antioxidative properties and ability to control antioxidant defenses based on our biochemical findings [81,82,83]. Moreover, their potential to considerably raise AChE levels and locomotory activities supported their neuroprotective characteristics. Synergistic and antibacterial action with conventional antibiotics against bacteria establishes their potential in biomedicine. As a result, it is stated that biosynthesis of Ag nanoparticles using the UD leaf fraction is an affordable, easy, and environmentally benign process that avoids the danger associated with the employment of toxic capping/reducing agents. Furthermore, this process may readily be upscaled for industrial uses to greatly increase the output of the nanoparticles, proving their viability for use in medicine.

## 4. Materials and Methods

### 4.1. Reagents and Chemicals

Gallic acid (C_7_H_6_O_5_), FC reagent (C_6_H_6_O), ABTS (C_18_H_18_N_4_O_6_S_4_), Ellman’s reagent (C_14_H_8_N_2_O_8_S_2_), sodium carbonate (Na_2_CO_3_), 1,1-diphenyl-2-picrylhydrazyl (C_18_H_12_N_5_O_6_), ascorbic acid (C_6_H_8_O_6_), aluminum chloride hexahydrate (AlCl_3_·6H_2_O), sulfuric acid (H_2_SO_4_), ferric chloride (FeCl_3_), bovine serum albumin, lysogeny broth, ethanol, and nutrient agar were purchased from Hi-Media (Mumbai, India). Acetylcholinesterase and silver nitrate were purchased from Sigma (Roeder mark, Germany). Using a spectrophotometer (Shimadzu UV-1601, Tokyo, Japan), calorimetric analysis was carried out.

### 4.2. Plant Collection and Identification

*Urtica dioica* (UD) young leaves were harvested before the plant started to produce seeds from apple orchards and local vegetable gardens in Srinagar, J&K, India. A taxonomist from the Botany Department at University of Kashmir (Srinagar, India) validated the identification of the medicinal plant.

#### 4.2.1. Preparation of UD Leaf Extraction

The harvested plant leaves were rinsed with tap water to remove surface contaminants and allowed to air dry for a week. Dry plant leaves were ground into a powder using a grinder after drying, and the powder was then sieved to obtain fine particles. Ten grams of the powder sample was combined with 100 mL of distilled water to create a 10% aqueous extract, which was then boiled at 95–100 °C for 10–15 min. Then, Whatman filter paper No. 1 was used to filter the extract. For future analysis, the extract was weighed and kept in an airtight container at 4 °C [84].

#### 4.2.2. Biosynthesis of Silver Nanoparticles (AgNPs)

For the biosynthesis of AgNPs, 2.5 mM silver nitrate (AgNO_3_) was added to the UD leaf extract (10 mL leaf extract in 90 mL silver nitrate). The solution was kept undisturbed in the dark for 2–3 days at room temperature (25 °C). After 2–3 days, the solution was centrifuged at 6000× *g* rpm for 20–25 min, and pellets were then heated in an oven at 50 °C for approximately 1–2 h, which were then extracted from the centrifuge tubes and converted for further testing [85].

#### 4.2.3. Radical Cation Decolorization Test Using the DPPH Assay

The 1,1-diphenyl-2-picrylhydrazyl (DPPH) assay was used to assess antioxidant potential [86]. For spectrophotometric analysis, a freshly made DPPH solution (11 mg in 50 mL methanol) was used. With the addition of methanol, the DPPH mixture was further diluted to achieve an optical density of 0.8–1. Each two mL of the DPPH mixture received a different concentration of plant components. A spectrophotometer (Shimadzu UV-1601, Tokyo, Japan) was used to detect the absorbance at 517 nm after incubation (30 min). DPPH was employed as a control, and methanol served as the blank. The experiments were conducted in triplicate. The equation below was used to estimate the percentage of free radical inhibition (% inhibition) of DPPH:

Inhibition of DPPH radicals % = [(A_control_ − A_test_)/A_control_] × 100

where A_control_ denotes the absorbance of the DPPH solution (used as a control), and A_test_ denotes the absorbance of UD and UD-AgNPs.

#### 4.2.4. Radical Cation Decolorization Test Using ABTS Assay

To evaluate the potential of UD-AgNPs to scavenge free radicals, the radical cation decolorization analyte (2,2′-azinobis 3-ethylbenzothiazoline-6-sulfonic acid) test was also utilized [87]. A potassium persulfate mixture (57 mg in 10 mL of methanol) and ABTS stock solution (36 mg in 10 mL of methanol) were combined in a ratio of 1:1 to produce free radical cations. The mixture was left at ambient temperature for 16 h in the dark. Methanol was used to further dilute the ABTS solution until it had an OD of 0.8–1. Samples at various compositions were diluted into the ABTS solution every two milliliters. Samples were analyzed at 745 nm following incubation (30 min). The formula below was used to estimate the percentage (% inhibition) of ABTS:

Inhibition of ABTS radicals % = [(A_control_ − A_test_)/A_control_] × 100

where A_control_ denotes the absorbance of the ABTS mixture (used as a control), and A_test_ denotes the absorbance of UD and UD-AgNPs.

#### 4.2.5. EC_50_ (Dose–Response Curve)

An EC_50_ is a quantitative assessment of the percentage of antibodies, medicine, or toxic substances that produces a half-maximal response between both the base point and maximal after a specified period. CompuSyn software (Version 1.0) was used to analyze the data (free radical scavenging properties and dose–response tests) to determine the potential of the samples. Lesser EC_50_ values suggest more radical scavenging ability [88].

#### 4.2.6. Determination of Total Phenolic Content (TPC)

The FC colorimetric technique was employed to evaluate the total phenolic content of UD and UD-AgNPs [89]. After five minutes, each sample was added to a 1:10 ratio of FC reagent (2.5 mL). The solution was then incubated (90 min) at room temperature before a UV/Vis spectrophotometer was used to measure the OD at 760 nm. The findings are expressed as milligrams of gallic acid equivalents (mg GAE/g) per gram of dry weight. Each sample was examined in triplicate.

$$C=\frac{c\;\times V}{m}$$

where “V” denotes the sample volume (UD and UD-AgNPs in microliters), “m” denotes the sample weight (grams), “C” is the sample total phenolic concentration (mg g^−1^ of GAE), and “c” stands for gallic acid (mg mL^−1^).

#### 4.2.7. Determination of Total Flavonoid Content (TFC)

Using the aluminum chloride colorimetric method, total flavonoids were measured [90]. The samples (UD and UD-AgNPs) were combined with 1.5 mL of methanol, 100 µL of AlCl₃ (10%), 0.1 mL of CH_3_CO_2_K (1 M), and double distilled water (2.8 mL). Then, the solution was left to react at ambient temperature (40 min) before the absorbance of the solution was measured at 415 nm. A calibration curve was made using quercetin. The number of total flavonoids was determined in the form of mg QE/g dry weight (quercetin equivalents). For each sample, measurements in triplicate were taken.

$$C=\frac{c\;\times V}{m}$$

where “C” stands for the sample’s total phenolic concentration (mg g^−1^), “c” for quercetin found in the sample as evaluated (calibration curve mg/mL), “V” for the sample’s volume (µL), and “m” for its weight (grams).

#### 4.2.8. UV-Visible Analysis

A spectrophotometer (Shimadzu UV-1601, Tokyo, Japan) was used to obtain the ultraviolet spectral data. The principal function of UV–visible spectroscopy is quantitative study, and it examines how light in the ultraviolet and visible spectrum is absorbed. Molecules or atoms can change from low to high energy levels owing to this radiation. Under certain circumstances, in solution, the number of molecules is inversely correlated with the amount of radiation absorbed. A correlation between absorption concentration and intensity was shown using spectral data. At the completion of the reaction, 1 mL of the suspension was taken from the purified samples (UD and UD-AgNPs) and sonicated at 6000 rpm for 10 min. UV–vis spectra were obtained between 200 and 800 nm at 1 nm intervals [91].

#### 4.2.9. FTIR Spectroscopic Analysis

One of the most effective methods for identifying the kinds of functional groups, chemical bonds, and molecular structures present in substances is Fourier-transform infrared spectroscopy (FTIR). The frequency of light that is absorbed is a good indication of the chemical bond, according to the detection patterns. The infrared absorption spectrum can be used to determine a molecule’s chemical bonds [92].

For FTIR investigation, aqueous extracts of UD and bio-fabricated UD-AgNPs were used. Ten milligrams of the sample were encased in a KBr pellet (100 mg) to create translucent sample discs. Next, FTIR spectroscopy was performed using a Perkin-Elmer Spectrophotometer on the sample material.

#### 4.2.10. FE-SEM with EDX Analysis

The topography, morphology, and size of the particles are all revealed in the FE-SEM image. The synthesized UD-AgNPs are uniform, agglomerated, and devoid of the other dominant phases. This is brought on by the individual UD-AgNPs’ high surface energy. With some variation, the size and structure of the UD-AgNPs are in good agreement with the XRD results. The purity and makeup of the UD’s green synthetic AgNPs are shown by the EDX spectrum. Strong signals from the Ag element and light signals from the O, C, and Cl elements can be seen in the EDX spectrum [93].

### 4.3. Fly Strain and Microorganisms

*Drosophila melanogaster* (Oregon R+, wild-type) were reared using the standard *Drosophila* diet, which includes agar, yeast, maize powder, sodium benzoate, propionic acid, and sugar [94]. The flies were kept at 24 °C, 68–70% relative humidity, and a 12-h cycle of dark and light. Freshly prepared pure *Escherichia coli* and *Pseudomonas putida* bacterial strains were prepared in sterile conditions at the laboratory of Lovely Professional University (Phagwara, India).

#### 4.3.1. Antimicrobial Activity by the Disc Diffusion Method

The antimicrobial activity of silver NPs was tested against *E. coli* and *P. putida* via the disc diffusion method by determining the zone of inhibition [95]. Different concentrations of AgNPs were taken (10, 20, and 40 µg/mL) for both *E. coli* and *P. putida*. Amoxicillin was used as a control for the comparison of bactericidal activity against AgNPs at a 20 µg/mL concentration. For this, the nutrient agar plate was prepared, and the pure culture of both bacteria was evenly spread in different plates under sterile conditions in laminar airflow. After that, four sterile filter paper discs were kept over the microbial spread. Then, the amoxicillin and AgNPs solutions were dropped over each disc at the abovementioned concentrations. After that, the plate was kept at 25 °C for over 24 h. Later, the zone of inhibition was measured.

#### 4.3.2. Treatment Schedule for *Drosophila*

Four groups were created in the experimental setup using third instar *D. melanogaster* larvae and adult flies. As a control, group I was fed the larvae standard *Drosophila* diet, while group II received food treated with silver nitrate. Group III was for UD alone, whereas group IV included the UD-AgNPs. Larvae were allowed to feed on regular food or food that had been subjected to treatments (AgNO_3_, UD, and UD-AgNPs) for 24 and 48 h, respectively. To measure their ability to climb and jump, the flies were treated for 5 days (120 h).

#### 4.3.3. Trypan Blue (Dye Exclusion) Assay

With a few minor modifications, Krebs and Feder’s (1997) description of dye exclusion was used [96]. This quick and easy procedure helps to distinguish between living and dead tissue cells. Cell death is detected in the larval gut. The larval gut was used to detect cell death. At the end of the treatment, 8 to 10 larvae were used, and their midguts were dissected. After that, the larvae were incubated for 13–15 min with a 0.2 mg/mL trypan blue dye solution in a 50.0 mM phosphate buffer saline (pH 7.4) solution and then washed three times with 0.1 M PBS. Using a stereomicroscope, the larvae were examined, and images were acquired.

#### 4.3.4. Preparation of Homogenate

Third instar larval midguts from control (normal/untreated), AgNO_3_, UD, and UD-AgNPs were dissected out and then crushed in ice-cold (0.1 M) of phosphate buffer (pH 7.4) including potassium chloride 0.15 M to obtain 10% cytosol/homogenate. After that, the samples were homogenized and centrifuged (4 °C) for 10 min at 12,000× *g* rpm. Then, the supernatant was used for various tests after being passed through a nylon membrane filter with 10 mm-sized pores [97].

#### 4.3.5. Acetylcholinesterase (AChE) Enzymatic Assay

As discussed previously Ellmann et al. (1961), AChE activity was evaluated. In brief, Ellman’s reagent (DTNB 10 mM), 78 mM acetylthiocholine iodide, and 0.01 mg protein (cytosolic sample) were all added to 0.1 M phosphate buffer (pH 8.0) to start the reaction. The shift in absorbance was tracked for 3 min at 412 nm. In terms of nmoles of substrate (hydrolyzed/min/mg protein), the enzyme activity was calculated [98].

#### 4.3.6. Protein Estimation

Bovine serum albumin (BSA) was used as the reference protein, and the Lowry et al. (1951) method was used to measure the protein concentration in the midgut of *D. melanogaster* homogenate [94].

#### 4.3.7. Evaluating Locomotor Impairments with a Climbing Assay

The climbing evaluation was carried out with specific modifications, as previously reported [99]. Twenty treated flies were maintained within a plastic vertical cylinder (20 cm long and 2 cm in width). Flies were counted if they passed the line (15 cm within 30 s) following tapping unless they arrived at the lowest section of the vials. The climbing count represents the typical extent of flies that passed the line (15 cm) out of all flies examined. The typical number of flies (over 15 cm n_top_ and under 15 cm n_bot_) are the counts, which are presented as a level of all flies (n_tot_). The findings are represented by the standard deviation of the counts obtained from three separate evaluations.

1/2[(n_tot_ + n_top_ − n_bot_)/n_tot_]

#### 4.3.8. Evaluating Locomotor Impairments with a Jumping Assay

Jumping activities were used to evaluate neuromuscular activation [100]. The frequency of locomotor function appears to influence the barrier for the jumping response. A vial marked 1–10 cm was filled with newly emerging flies one at a time, and the distance each fly leaped from the vial’s bottom was recorded. The jumping behavior was identified as the estimated value of jumps completed over 5 repetitions. Each set of 100 flies was used in five replicates.

### 4.4. Data Analysis

Using two-way ANOVA and Tukey’s test in the SPSS statistical analysis program, colorimetric data presented as the mean ± SEM and *n* = 3 were examined for significant variations. A *p* value < 0.05 or less was used to determine the significance.

## 5. Conclusions

Current research shows that the antiradical and antioxidant properties of *U. dioica* and biosynthesized Ag nanoparticles are statistically significant (*p* < 0.001). In this study, *U. dioica* was used for the green synthesis of AgNPs, the leaf extract of which acts as a reducing agent for the reduction of Ag^+^ ions from AgNO_3_. The existence and purity of silver present in the AgNPs can be checked and confirmed with the help of UV–vis, FTIR, SEM, and EDS. The antioxidant activities of AgNPs are normally estimated using the ABTS and DPPH free radical scavenging methods. During the antimicrobial screening test [100], the zone of inhibition proved that the silver nanoparticles (AgNPs) produced in this process are efficient for antimicrobial activity against pathogenic bacteria. Administration of UD-AgNPs to *Drosophila* has the potential to alleviate oxidative stress due to its antioxidative activities and ability to control antioxidant defenses. Moreover, their neuroprotective characteristics were evaluated by their ability to dramatically enhance cellular uptake, AChE levels, and locomotory activities. The green synthesis of silver nanoparticles could be of immense use in the future against various ailments, including cancer and neurodegeneration. The nanoparticles can be used in medicines, and with the help of technology, these nanoparticles can be programmed to cure specific diseases by targeting their specific sites.

## Figures and Tables

**Table 2:** Using DPPH and ABTS assays, various models were used to estimate the predicted EC_50_ (mg/mL) of UD and UD-AgNPs.

S. No.	Assays	Samples	EC50 (mg/mL)
1.	DPPH	UD	0.12
2.		UD-AgNPs	0.05
3.	ABTS	UD	0.22
4.		UD-AgNPs	0.07

**Table 3:** TPC and TFC results for UD and UD-AgNPs are shown as the mean ± SD.

S. No.	Assays	Concentration (mg/mL)	*Urtica dioica* (UD)	Biosynthesized AgNPs of UD
1.	TFC (mg (QE)/g)	0.1	6.391	8.137
2.		0.2	9.168	12.210
3.		0.3	14.62	17.945
4.	TPC (mg (GAE)/g)	0.1	21.375	29.916
5.		0.2	28.625	35.833
6.		0.3	33.125	40.00

**Table 4:** Antimicrobial effect of AgNPs in *Escherichia coli* and *Pseudomonas putida*.

Name of Bacteria	Concentration of AgNPs (µg/mL)	Zone of Inhibition (in mm)
*E. coli*	10	3 ± 0.2
	20	7 ± 0.44
	40	8 ± 0.2
	Antibiotics (20 µg/mL)	10 ± 1.73
*P. putida*	10	4 ± 0.26
	20	5 ± 0.1
	40	8 ± 0.2
	Antibiotics (20 µg/mL)	11 ± 1.0

Values are given as the mean ± SD, *n* = 3.

## Data Availability

Data are contained within the article.
